# The association between hemoglobin-to-red blood cell distribution width ratio and 28-day mortality in epidural hemorrhage: a cohort study

**DOI:** 10.3389/fneur.2025.1534098

**Published:** 2025-07-08

**Authors:** Hua Liu, Hong Huang, Jinrong Wang, Wenming Wang, Min Ruan, Jiangang Liu

**Affiliations:** ^1^Department of Neurosurgery, Affiliated Kunshan Hospital of Jiangsu University, Kunshan, China; ^2^Kunshan Biomedical Big Data Innovation Application Laboratory, Kunshan, China; ^3^Department of Neurosurgery, Kunshan Hospital of Traditional Chinese Medicine, Kunshan Affiliated Hospital of Nanjing University of Chinese Medicine, Kunshan, China; ^4^Department of Neurosurgery, The First Affiliated Hospital of Soochow University, Suzhou, China

**Keywords:** epidural hematoma, hemoglobin-to-red blood cell distribution width ratio, 28-day mortality, ICU, eICU-CRD

## Abstract

**Background:**

This study leverages the eICU collaborative research database (eICU-CRD) to investigate the relationship between the hemoglobin-to-red blood cell distribution width ratio (HRR) and 28-day mortality in patients with epidural hematoma (EDH).

**Methods:**

A total of 2,161 patients admitted between 2014 and 2015 with EDH were selected. Data included demographics, medical history, and laboratory tests. HRR was calculated and stratified into quartiles. Covariates included Glasgow Coma Scale (GCS), HDL, TG, hospital time, ICU time, LDL, age, BMI, gender, coma status, race, and medical conditions like COPD, CHF, and diabetes. Non-normal data distributions were analyzed using Kruskal-Wallis and chi-square tests, with logistic regression to explore the association of HRR and 28-day mortality.

**Results:**

Higher HRR quartiles correlated with lower 28-day mortality (*p* = 0.024) and higher healthy discharge rates (*p* = 0.013). Univariate logistic analysis showed age positively associated with mortality (OR = 1.011, 95% CI: 1.004–1.018), while GCS, ICU time, hospital time, and HRR were negatively associated. Adjusted models confirmed an inverse relationship between HRR and mortality, with the fourth quartile showing a 40% reduced probability of mortality. Linear regression models indicated a 72% reduction in mortality risk per unit HRR increase and a critical HRR value of 1.12 for significant risk reduction.

**Conclusion:**

HRR is significantly associated with 28-day mortality in EDH patients, with higher HRR values correlating with improved survival. ICU time also showed a correlation with reduced mortality, particularly up to a critical point.

## Introduction

Epidural hematoma (EDH) is one of the common diseases in neurosurgery (1–3). It refers to the collection of blood that forms a hematoma within the potential space between the dura mater and the inner table of the skull (4, 5). It is usually caused by head trauma that results in skull fractures or temporary deformation of the skull, which then tears the meningeal arteries or venous sinuses located in the grooves, leading to bleeding (6–9). EDH is more common in adolescents and young adults, especially males between the ages of 15 and 50, with a relatively lower incidence in infancy (10, 11). The incidence of EDH accounts for 1 to 4% of craniocerebral injuries, 2 to 3% of closed craniocerebral injuries, and 25 to 30% of all intracranial hematomas (12, 13). The clinical manifestations of EDH typically include disturbances of consciousness, increased intracranial pressure, and neurological signs (14, 15). Disturbances of consciousness may present as a pattern of coma-arousal-coma, increased intracranial pressure may be accompanied by symptoms such as headache, nausea, and severe vomiting, and neurological signs may include central facial paralysis and hemiplegia, severe cases may result in disability or death (16, 17). The mortality rate of EDH ranges from 10 to 25%, with the main cause of death not being the hematoma itself, but rather the secondary damage to the brainstem caused by the formation of brain herniation (18, 19). The challenge in treating EDH lies in early diagnosis and timely management, as any delay can significantly increase the risk of mortality and disability. In light of this challenging clinical issue, there is an urgent need for a predictor of mortality in EDH to guide clinical diagnosis and treatment.

HRR is considered an inflammatory marker associated with the incidence of various diseases (20–22), such as diabetes (23), non-small cell lung cancer (24), and atrial fibrillation (25). HRR primarily consists of two hematological indicators: hemoglobin (HB) and red blood cell distribution width (RDW) (26, 27). HB mainly reflects the degree of anemia, while RDW indicates the heterogeneity of red blood cell volume and is indirectly used for diagnosing anemia (28, 29). Previous studies have shown that HRR undergoes dynamic changes in response to disease stimuli, and its level variations can serve as a sensitive indicator of inflammation (30, 31). Although HRR, as an inflammatory marker, is related to many diseases, the clinical relationship between HRR and the 28-day mortality rate in patients with EDH remains unclear.

Given that the prognosis of EDH may be related to inflammation, there might be a unique link between HRR and the 28-day mortality rate in EDH. However, current research is unclear about the relationship between HRR and the prognosis of EDH. Therefore, in this study, we extracted data on HRR and EDH from the eICU-CRD between 2014 and 2015, attempting to explore whether there is a specific correlation between HRR and the 28-day mortality rate in patients with EDH.

## Materials and methods

### Study population and study design

**Figure 1:**
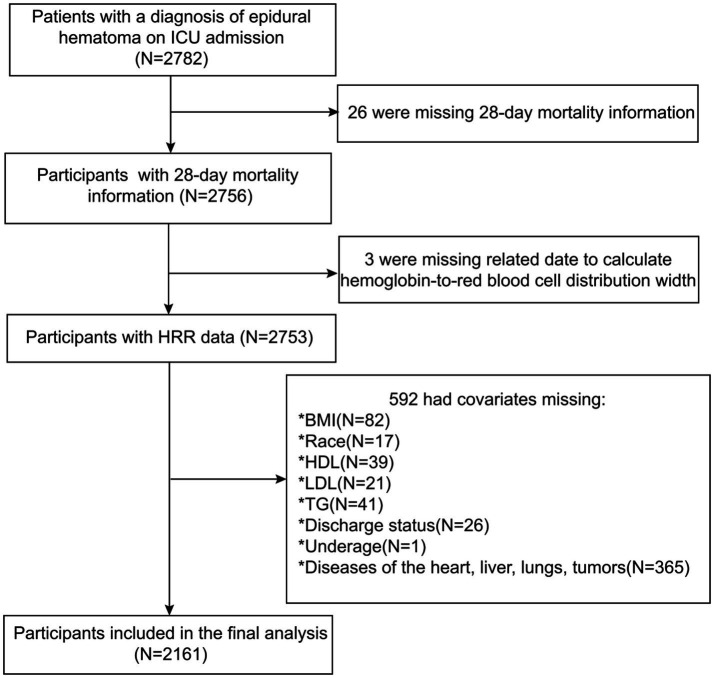
Research flowchart.

The eICU Collaborative Research Database (eICU-CRD) is a large, multicenter clinical database comprised of data from intensive care units (ICUs) across numerous hospitals in the United States. The eICU-CRD compiles a wealth of information on admitted patients, including age, gender, admission diagnoses, admission and discharge times, 28-day mortality rates, and various laboratory tests and scale scores, providing clinicians with authentic and detailed research materials for their scientific endeavors. Additionally, the project has been approved by the Massachusetts Institute of Technology Institutional Review Board, thus eliminating the need for additional ethical clearance for this study. Figure 1 provides a detailed depiction of the participant selection process for this research. Initially, we identified patients admitted between 2014 and 2015 with a diagnosis of EDH from the eICU-CRD, totaling 2,782 individuals. The EDH in our study is caused by trauma, which is the most common etiology for EDH. The epidural hematoma is isolated and not in patients with polytrauma, without traumatic subarachnoid hemorrhage, subdural hematoma or intracerebral hemorrhage. Given that all cases of EDH in our study are trauma-induced, they are classified as acute. We also collected their general information (e.g., age, gender), past medical history (e.g., COPD, CHF), hospitalization details (e.g., admission and discharge times, hospital status), and necessary laboratory tests. Subsequently, due to incomplete data for some patients in the eICU-CRD, the first step was to exclude those lacking information on 28-day mortality, resulting in the exclusion of 26 participants. Next, due to missing data required for calculating HRR, we removed three participants. To further enhance the credibility of the study, we continued to screen for potential confounding factors, leading to the exclusion of an additional 592 participants who lacked information on these factors. Specifically, 82 individuals were missing BMI data, 17 lacked racial data, 39 had unknown HDL levels, 21 did not have LDL data, 26 did not provide discharge status information, 41 did not have TG measurements, one was a minor, and 365 did not provide related medical histories of heart, liver, lung, or cancer. After these exclusions, a total of 2,161 participants were ultimately included in the study.

### Measurement of epidural hemorrhage

The diagnosis of epidural hemorrhage is primarily based on admission diagnoses from the eICU-CRD, while excluding cases of spontaneous intracerebral hemorrhage, bleeding related to hematological disorders, hemorrhage caused by cerebral vascular malformations, and hemorrhage resulting from the rupture of cerebral aneurysms.

### Definition of the HRR

The hemoglobin-to-red blood cell distribution width ratio (HRR) is calculated by dividing hemoglobin (HB) levels by the red blood cell distribution width (RDW), utilizing laboratory data from the eICU-CRD. These data are all the results of the first blood draw at admission, which is more conducive to controlling confounding factors and strengthening the reliability of the research results. For subsequent analysis, HRR is stratified into quartiles. And the HRR data in our study were collected at the time of the patient’s initial admission.

### Covariates

Building on previous related research, this study included Glasgow Coma Scale (GCS), high-density lipoprotein (HDL), triglycerides (TG), hospital time, ICU time, low-density lipoprotein (LDL), age, body mass index (BMI), gender, coma status, race, chronic obstructive pulmonary disease (COPD), chronic heart failure (CHF) and diabetes as covariates. BMI is calculated from anthropometric data, specifically height (HT in meters, m) and weight (WT in kilograms, kg), using the formula BMI = WT/(HT)^2^. The assessment of coma status is based on the Glasgow Coma Scale (GCS) score. The GCS is a standardized scoring system used to evaluate a patient’s level of consciousness and depth of coma. It is widely utilized in the medical field, particularly in emergency medicine, neurosurgery, and intensive care. The GCS score consists of three components: eye opening, verbal response, and motor response, each ranging from 1 to 6 points, with higher scores indicating better responsiveness. In this study, a GCS score below 8 is considered to be in a state of coma.

### Statistical analysis

In this study, we conducted thorough data processing and quality control. Given the non-normal distribution of the data, continuous variables are presented as medians with interquartile ranges (Q1, Q3), while categorical variables are expressed as counts and percentages. The Kruskal-Wallis test for continuous variables and the chi-square test for categorical variables were used to compare 28-day mortality. A *p*-value less than 0.05 was considered to indicate a statistically significant difference.

We explored the relationship between HRR and 28-day mortality of EDH using logistic regression analysis appropriate for the study design. The robustness of the model was progressively strengthened by adjusting for different covariates. Specifically, Model 1 is the most basic model without adjustment for any variables. Model 2 is adjusted for race, age, and gender. Finally, Model 3 includes adjustments for potential confounding factors such as gender, age, race, BMI, HDL, TG, LDL, GCS, hospital time, ICU time, tumor metastasis, liver failure, diabetes, COPD, CHF, and coma, thereby further enhancing the robustness of the model.

## Results

### Demographic and initial characteristics of participants

The study selected 2,161 adult Americans from the eICU-CRD database between 2014 and 2015, with a higher number of females, totaling 1,104 individuals, accounting for approximately 51.09% of the study population. Within this cohort, 401 individuals died in the ICU within 28 days due to EDH, representing 18.56% of the sample size. The majority of participants were identified as Caucasian, with a total of 1,576 individuals, constituting 72.93% of the total study cohort. Table 1 summarizes the demographic and baseline characteristics of the study subjects, stratified by HRR quartiles. Individuals with higher HRR showed a positive correlation with healthy discharge rates (*p* = 0.013), and a negative correlation with 28-day mortality due to EDH (*p* = 0.024). Additionally, there were differences in HB levels and RDW levels in the blood indicators of this patient group (*p* < 0.001). However, there were no significant differences in age, gender, race, HDL, LDL, TG, BMI, COPD, CHF, diabetes, and GSC across the HRR quartiles (*p* > 0.05).

**Table 1:** Characteristics of the participants categorized by HRR.

HRR	Q1 (0.41–1.03)	Q2 (1.04–1.14)	Q3 (1.14–1.23)	Q4 (1.23–1.54)	*p*-value
*N*	540	540	540	541	
Age, years	69.00 (20.00–89.00)	67.00 (18.00–89.00)	66.50 (18.00–89.00)	67.00 (21.00–89.00)	0.329
HDL, mmol/L	1.29 (0.54–4.60)	1.27 (0.36–3.13)	1.29 (0.59–3.26)	1.32 (0.54–4.29)	0.395
TG, mmol/L	1.21 (0.34–7.52)	1.11 (0.23–7.90)	1.09 (0.27–17.64)	1.13 (0.16–11.21)	0.078
LDL, mmol/L	2.90 (0.78–7.50)	2.81 (0.62–6.05)	2.90 (0.83–5.46)	2.85 (0.62–6.28)	0.412
BMI, kg/m^2^	26.62 (12.60–61.69)	26.47 (11.35–56.74)	26.52 (14.81–66.23)	26.93 (10.21–71.04)	0.283
RDW, %	13.40 (11.10–28.40)	12.70 (11.40–15.30)	12.40 (11.00–15.90)	12.20 (10.60–13.90)	<0.001
HB, g/dL	12.80 (8.20–17.30)	13.90 (12.10–16.60)	14.70 (12.60–19.20)	15.80 (13.80–19.70)	<0.001
GCS	14.00 (3.00–15.00)	13.00 (3.00–15.00)	14.00 (3.00–15.00)	14.00 (3.00–15.00)	0.553
Hospital time, days	5.39 (0.11–54.13)	5.77 (0.03–270.08)	5.92 (0.10–146.09)	5.89 (0.05–90.87)	0.182
ICU time, days	2.27 (0.06–37.68)	2.37 (0.02–32.57)	2.12 (0.00–38.18)	2.20 (0.05–42.47)	0.979
Gender, *n* (%)					0.567
Male	272 (50.37)	252 (46.67)	261 (48.33)	272 (50.28)	
Female	268 (49.63)	288 (53.33)	279 (51.67)	269 (49.72)	
Race, *n* (%)					0.628
Caucasian	395 (73.15)	402 (74.44)	383 (70.93)	396 (73.20)	
African American	66 (12.22)	62 (11.48)	80 (14.81)	81 (14.97)	
Hispanic	35 (6.48)	34 (6.30)	36 (6.67)	22 (4.07)	
Asian	29 (5.37)	24 (4.44)	30 (5.56)	24 (4.44)	
Native American	3 (0.56)	4 (0.74)	2 (0.37)	2 (0.37)	
Other race	12 (2.22)	14 (2.59)	9 (1.67)	16 (2.96)	
Tumor metastasis, *n* (%)					0.719
No	528 (97.78)	530 (98.15)	530 (98.15)	534 (98.71)	
Yes	12 (2.22)	10 (1.85)	10 (1.85)	7 (1.29)	
Liver failure, *n* (%)					0.611
No	535 (99.07)	538 (99.63)	535 (99.07)	538 (99.45)	
Yes	5 (0.93)	2 (0.37)	5 (0.93)	3 (0.55)	
Diabetes, *n* (%)					0.097
No	471 (87.22)	457 (84.63)	478 (88.52)	454 (83.92)	
Yes	69 (12.78)	83 (15.37)	62 (11.48)	87 (16.08)	
COPD, *n* (%)					0.096
No	535 (99.07)	525 (97.22)	529 (97.96)	534 (98.71)	
Yes	5 (0.93)	15 (2.78)	11 (2.04)	7 (1.29)	
CHF, *n* (%)					0.464
No	528 (97.78)	533 (98.70)	532 (98.52)	535 (98.89)	
Yes	12 (2.22)	7 (1.30)	8 (1.48)	6 (1.11)	
Coma, *n* (%)					0.340
No	402 (74.44)	415 (76.85)	412 (76.30)	428 (79.11)	
Yes	138 (25.56)	125 (23.15)	128 (23.70)	113 (20.89)	
Discharge status, *n* (%)					0.013
No	413 (76.48)	444 (82.22)	447 (82.78)	451 (83.36)	
Yes	127 (23.52)	96 (17.78)	93 (17.22)	90 (16.64)	
28-day mortality, *n* (%)					0.024
No	416 (77.04)	445 (82.41)	448 (82.96)	451 (83.36)	
Yes	124 (22.96)	95 (17.59)	92 (17.04)	90 (16.64)	

### Univariate logistic analyses revealed the associations between multiple variables with 28-day mortality in EDH

As shown in Table 2, in the univariate analysis, age (OR = 1.011, 95% CI: 1.004–1.018) were positively associated with 28-day mortality due to EDH (*p* = 0.003). And GCS (OR = 0.692, 95% CI: 0.670–0.715), ICU time (OR = 0.940, 95% CI: 0.915–0.967), hospital time (OR = 0.835, 95% CI: 0.808–0.862), HRR (OR = 0.296, 95% CI: 0.154–0.568) were negatively associated with 28-day mortality in EDH (*p* < 0.001).

**Table 2:** Weighted univariate logistic analyses between variables and epidural hematoma 28-day mortality (odds ratios, 95% confidence intervals).

Variables	Univariate analysis (crude model)		
	Median ± SD	OR (95% CI)	*p*-value
Age	65.923 ± 15.870	1.011 (1.004, 1.018)	0.003
BMI	27.762 ± 6.959	0.988 (0.972, 1.004)	0.142
TG	1.414 ± 1.054	1.096 (0.998, 1.203)	0.055
LDL	2.913 ± 0.887	0.981 (0.868, 1.109)	0.763
HDL	1.355 ± 0.393	0.987 (0.748, 1.301)	0.925
GCS	11.394 ± 4.247	0.692 (0.670, 0.715)	<0.001
ICU time	4.303 ± 5.368	0.940 (0.915, 0.967)	<0.001
Hospital time	8.552 ± 11.428	0.835 (0.808, 0.862)	<0.001
HRR	1.121 ± 0.161	0.296 (0.154, 0.568)	<0.001

### The relationship between HRR quartiles and 28-day mortality in EDH

Referencing Table 3, an increase in the HRR quartiles is associated with a decreased risk of 28-day mortality in EDH. The unadjusted model reveals a negative correlation between HRR quartiles and 28-day mortality in EDH (Model 1). After partial adjustment in Model 2, this correlation remains significantly meaningful. Taking into account all potential confounding factors, Model 3 shows that compared to the reference quartile (Q1), the odds ratios (OR) for 28-day mortality in EDH associated with the second (Q2), third (Q3), and fourth (Q4) quartiles are approximately 0.70 (95% CI: 0.50, 0.97), 0.64 (95% CI: 0.45, 0.92), and 0.60 (95% CI: 0.41, 0.89) respectively (*p* < 0.001). Furthermore, under strict control of potential confounding variables, for example, at the HRR Q4 level, an increase of one unit in HRR is associated with a 40% reduction in the probability of 28-day mortality in EDH.

**Table 3:** Multivariate Cox regression analysis of HRR with epidural hematoma 28-day mortality.

	HRR quartiles				*p* for trend
	Q1	Q2	Q3	Q4	
	0.41–1.03	1.04–1.14	1.14–1.23	1.23–1.54	
	OR (95% CI)	OR (95% CI)	OR (95% CI)	OR (95% CI)	
28-day mortality	124 (22.96%)	95 (17.59%)	92 (17.04%)	90 (16.64%)	
Model 1	Reference	0.68 (0.51, 0.90)	0.69 (0.51, 0.93)	0.67 (0.49, 0.91)	<0.001
Model 2	Reference	0.73 (0.54, 0.98)	0.69 (0.51, 0.94)	0.68 (0.50, 0.92)	<0.001
Model 3	Reference	0.70 (0.50, 0.97)	0.64 (0.45, 0.92)	0.60 (0.41, 0.89)	<0.001

Values are presented as weighted odds ratios (ORs), 95% confidence interval (95% CI), and *p*-value.

Model 1 adjusted for none.

Model 2 adjusted for gender, age, and race.

Model 3 adjusted for gender, age, race, BMI, HDL, TG, LDL, GCS, hospital time, ICU time, tumor metastasis, liver failure, diabetes, COPD, CHF, and coma.

**Figure 2:**
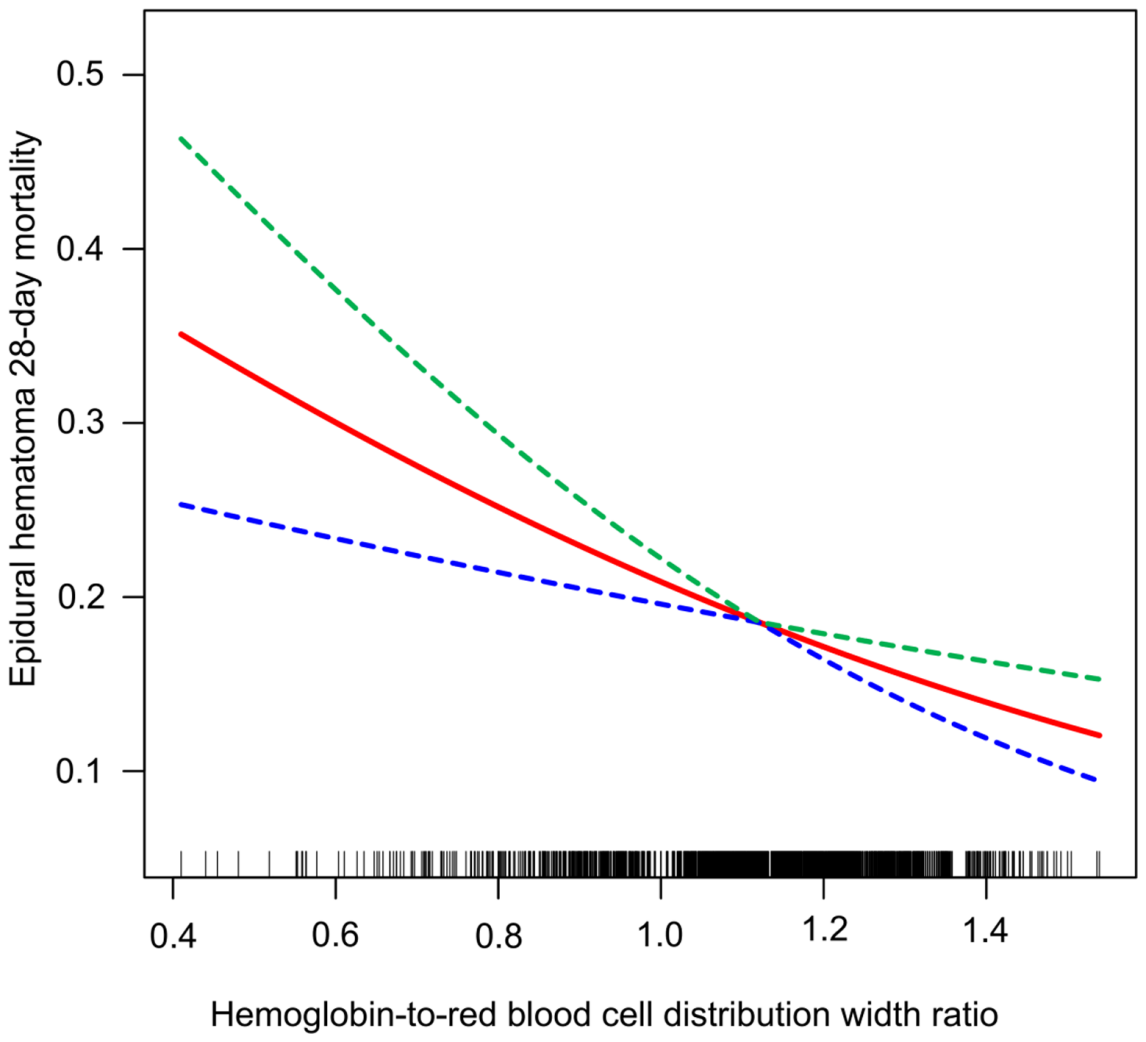
Relationship between HRR and the 28-day mortality in EDH patients.

Utilizing smooth curve fitting and threshold effect analysis, we assessed the potential linear correlation between HRR and 28-day mortality in EDH. As shown in Figure 2, the study results indicate a substantial linear association between these variables. Table 4 details the outcomes of the standard linear regression model, demonstrating a 72% reduction in PAD risk for each unit increase in HRR (*p* = 0.0012). Two piecewise linear regression models identified a critical point at an HRR value of 1.12. For HRR values equal to or below 1.12, each unit increase in HRR resulted in a 80% reduction in the odds of PAD (OR = 0.20, 95% CI = 0.06–0.66, *p* = 0.0078). When HRR exceeds 1.12, additional unit increases in HRR show no significant correlation with 28-day mortality in EDH. Thus, combining these findings, there is a clear linear relationship between HRR and 28-day mortality in EDH.

**Table 4:** Threshold and saturation effect analysis of HRR on epidural hematoma 28-day mortality.

Outcomes	Epidural hematoma 28-day mortality	
	OR (95% CIs)	*p*-value
Model A		
(Fitting model by standard linear regression)	0.28 (0.13, 0.60)	0.0012
Model B		
(Fitting models by two-piecewise linear regression)		
Infection point	1.12	
<Infection point	0.20 (0.06, 0.66)	0.0078
>Infection point	0.52 (0.08, 3.44)	0.4959
*p* for log-likelihood ratio test	0.486	

The two-piecewise regression models were adjusted for gender, age, race, BMI, HDL, TG, LDL, GCS, hospital time, ICU time, tumor metastasis, liver failure, diabetes, COPD, CHF and coma.

OR, odds ratios; 95% CI, 95% confidence interval.

### The relationship between ICU time and 28-day mortality in EDH

**Figure 3:**
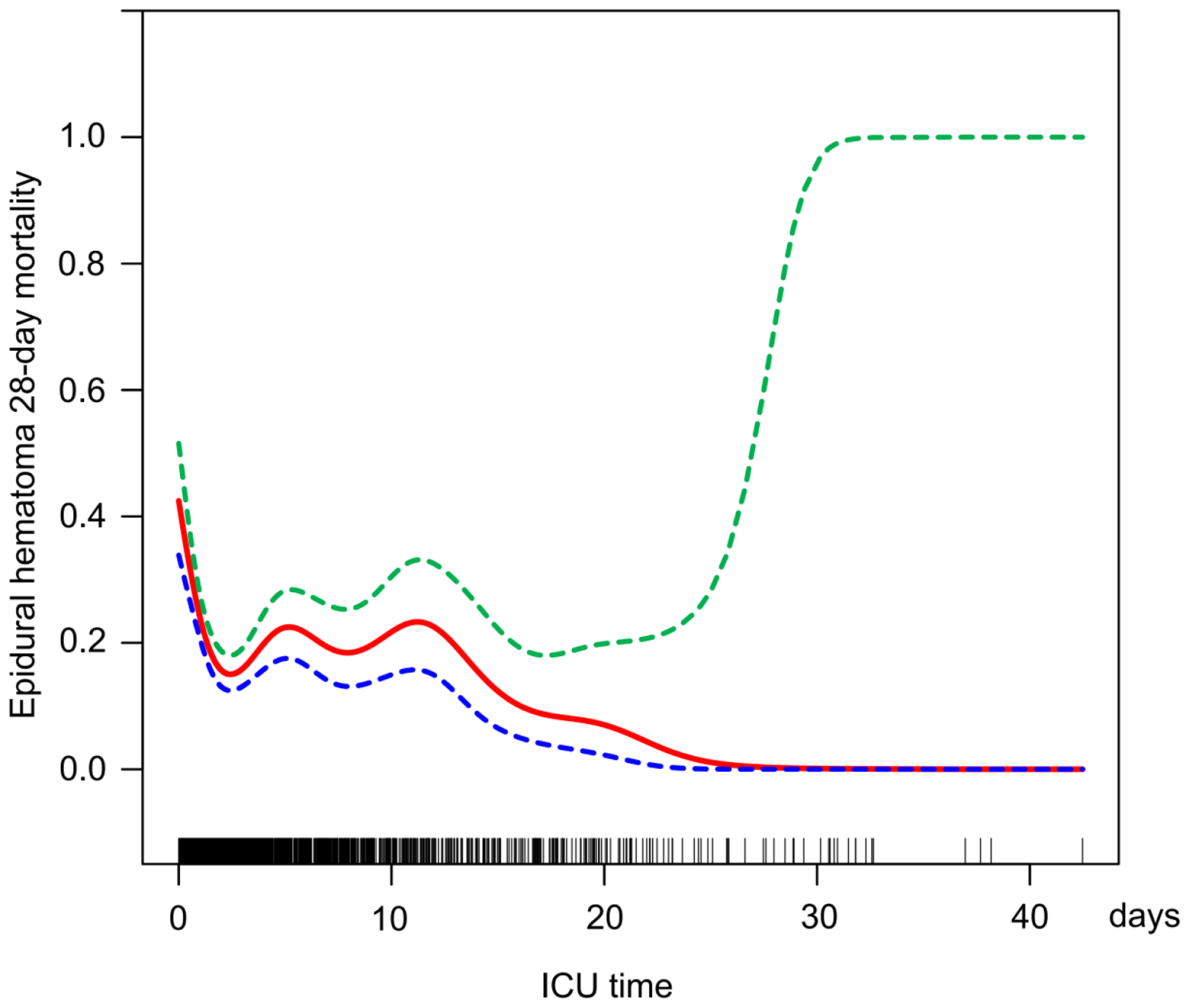
Relationship between HRR and the 28-day mortality in EDH patients.

Additionally, this study employed the same methodology to assess the potential linear correlation between ICU time and 28-day mortality in EDH. As depicted in Figure 3, the study results indicate a certain degree of correlation between these variables. Table 5 details the outcomes of the standard linear regression model, showing an 8% reduction in 28-day mortality in EDH for each unit increase in ICU time (*p* = 0.0012). Two piecewise linear regression models identified a critical point at an ICU time value of 0.91 days. For ICU stays equal to or less than 0.91 days, each unit increase in ICU time resulted in an 81% decrease in 28-day mortality in EDH (OR = 0.19, 95% CI = 0.10–0.36, *p* < 0.0001). When ICU time exceeded 0.91 days, additional unit increases in ICU time showed a less pronounced trend in reducing 28-day mortality in EDH (OR = 0.95, 95% CI = 0.91–0.98, *p* = 0.0045), and the log-likelihood ratio test yields a *p* value less than 0.001. Therefore, combining these findings, the extension of ICU stay contributes to the reduction of 28-day mortality in EDH.

**Table 5:** Threshold and saturation effect analysis of ICU time on epidural hematoma 28-day mortality.

Outcomes	Epidural hematoma 28-day mortality	
	OR (95% CIs)	*p*-value
Model A		
(Fitting model by standard linear regression)	0.92 (0.89, 0.96)	<0.0001
Model B		
(Fitting models by two-piecewise linear regression)		
Infection point	0.91	
<Infection point	0.19 (0.10, 0.36)	<0.0001
>Infection point	0.95 (0.91, 0.98)	0.0045
*p* for log-likelihood ratio test	<0.001	

The two-piecewise regression models were adjusted for gender, age, race, BMI, HDL, TG, LDL, GCS, hospital time, ICU time, tumor metastasis, liver failure, diabetes, COPD, CHF, and coma.

OR, odds ratios; 95% CI, 95% confidence interval.

## Discussion

EDH is a critical condition in neurosurgery, characterized by the accumulation of blood between the dura mater and the skull, typically resulting from head trauma (32–34). Our study, leveraging data from the eICU-CRD between 2014 and 2015, aimed to investigate the correlation between the hemoglobin-to-red blood cell distribution width ratio (HRR) and the 28-day mortality rate in patients with EDH. Our findings suggest that HRR may serve as a significant predictor of mortality in this patient population.

Zangbar’s et al. (35) study collected clinical data from 76 patients with traumatic brain injury (TBI) between 2010 and 2012, using neurosurgical intervention (NI) as the outcome measure. The average age of this cohort was 20.6 ± 15.2 years, with 68.4% being male, and a median Glasgow Coma Scale (GCS) score of 15 (13–15). The study found that patients who underwent NI had longer hospital stays (*p* = 0.02) and longer stays in the intensive care unit (*p* = 0.05). The incidence of isolated epidural hematoma (EDH) was lower in patients with blunt TBI. Compared to patients without NI, those with isolated EDH who received NI had longer hospitalizations. Another study collected surgical data from 92 patients with acute traumatic EDH treated between 2007 and 2018. Computed tomography (CT) was used to locate the position of EDH, categorized as lateral or medial, with the specific site of bleeding determined by neurosurgeons during surgery. The study aimed to explore the relationship between the CT-identified location of EDH and the source of bleeding. The results revealed that arterial bleeding was the cause of EDH in 63.4% of lateral EDH cases and 9.2% of medial EDH cases (*p* < 0.0001). Among the cases primarily managed with surgical treatment for EDH, 65.3% of patients had an arterial source of bleeding (*p* < 0.0001). This indicates that the location of EDH is correlated with the source of bleeding (36). The relationship between HRR and mortality in EDH has not been extensively studied (37–39), and our research contributes to the understanding of this association. We discovered a substantial linear relationship between HRR and 28-day mortality in EDH, indicating that higher HRR values are associated with a reduced risk of mortality. This is consistent with the hypothesis that HRR, as an inflammatory marker, could reflect the body’s response to the injury and subsequent inflammation caused by EDH.

Our results align with previous studies that have implicated HRR in various disease outcomes, including diabetes, non-small cell lung cancer, and atrial fibrillation. The negative correlation between HRR and 28-day mortality in EDH suggests that HRR may be a protective factor, potentially due to its role in the inflammatory response. This is further supported by the finding that higher HRR quartiles were associated with a decreased risk of mortality, with the most significant reduction observed in the highest quartile.

The strength of our study lies in its comprehensive approach, adjusting for multiple confounding factors to ensure the robustness of our findings. However, we acknowledge the limitations inherent in our study design. The retrospective nature of the study and the reliance on data from a single database may introduce biases that could affect the generalizability of our results. Additionally, while we controlled for numerous covariates, there may be other unmeasured factors that could influence the relationship between HRR and mortality in EDH. However, the clinical implications of our findings are significant. If HRR can be confirmed as a predictor of mortality in EDH, it could guide clinical decision-making, potentially leading to more targeted and effective treatment strategies. Early identification of patients at higher risk of mortality could facilitate timely interventions, improving patient outcomes.

In conclusion, our study provides novel insights into the role of HRR in EDH and its potential as a prognostic marker for 28-day mortality. Further prospective studies are warranted to validate our findings and explore the underlying mechanisms that link HRR to mortality in EDH. This research could pave the way for the incorporation of HRR into clinical practice as a tool for risk stratification and personalized treatment planning.

## Conclusion

The study reveals a significant linear relationship between higher HRR values and reduced 28-day mortality in patients with EDH, with a critical threshold identified at an HRR of 1.12. Additionally, extended ICU stays are associated with decreased 28-day mortality, suggesting the importance of intensive care duration in patient outcomes.

## Data Availability

The original contributions presented in the study are included in the article/supplementary material, further inquiries can be directed to the corresponding authors.
